# LncRNA PEG11as silencing sponges miR-874-3p to alleviate cerebral ischemia stroke via regulating autophagy *in vivo* and *in vitro*

**DOI:** 10.18632/aging.204140

**Published:** 2022-06-24

**Authors:** Xiamin Hu, Fuyun Ma, Zhongliang Cheng, Suyou Zeng, Ruling Shen, Xuan Li, Junqi Hu, Zhigang Jin, Jinping Cheng

**Affiliations:** 1 College of Pharmacy, Shanghai University of Medicine and Health Sciences, Shanghai, China; 2 Wuhan University of Science and Technology Affiliated Wuhan Resources and Wisco General Hospital, Wuhan, Hubei, China; 3 Shanghai Laboratory Animal Research Center, Shanghai, China; 4 University of California, San Diego, CA 92093, USA

**Keywords:** PEG11as, miR-874-3p, ATG16L1, autophagy, cerebral ischemia/reperfusion

## Abstract

Long non-coding RNAs (lncRNAs) are reportedly involved in the regulation of physiological and pathophysiological processes. However, the potential role of lncRNAs in stroke remains largely undefined. Here, RNA-Seq analysis of lncRNAs found that the lncRNA PEG11as (PEG11as) levels were significantly increased in ischemic brain tissue in a transient middle cerebral artery occlusion/reperfusion (tMCAO/R) mouse model of stroke. To explore the role of PEG11as in stroke, the lentivirus containing PEG11as silencing construct(siRNA-PEG11as) was microinjected intracerebroventricularly into male or transfected to N2a cells and then exposed to tMCAO/R or oxygen-glucose deprivation/reoxygenation (OGD/R). Knockdown of PEG11as expression significantly reduced infarct volume, alleviated neuronal deficits and inhibited neuronal apoptosis in tMCAO/R mice. Mechanistically, as an endogenous microRNA-874-3p (miR-874-3p) sponge, PEG11as silencing inhibited miR-874-3p activity, resulting in downregulation of ATG16L1 expression and subsequent inhibition of neuronal apoptosis by regulating autophagy. Overall, the results of this current study indicate that PEG11as is involved in the pathophysiology of cerebral ischemia, thus providing translational evidence that PEG11as can be envisioned as a novel biomarker or/and therapeutic target for stroke.

## INTRODUCTION

Stroke remains a serious problem with immense socioeconomic impact worldwide despite recent therapeutic advances [1], and aging is the most robust non-modifiable risk factor [2]. Acute ischemic stroke is the most common stroke type which accounts for over 80% of all stroke [3]. Accident resulted in a lack of blood supply to the brain tissue and sudden death of brain cells from lack of oxygen during a stroke. Thrombolytic therapy is a recognized to be clinically effective treatment; However, the narrow treatment time window and safety issues limit its clinical application. Therefore, it is critical to further clarify the intricate mechanisms and explore new therapeutic strategies.

Long non-coding RNAs (lncRNAs) do not encode protein and they are greater than 200 nucleotides in length [4], and they have become a major source of biomarkers and therapeutic targets [5, 6]. LncRNAs regulate gene expression [7], which is closely related to a variety of neurological diseases, including ischemic stroke [8]. A growing body of research suggests that lncRNA MALAT1 could inhibit autophagy by regulating microRNA-30 in ischemic stroke, leading to inhibit neuronal apoptosis [9, 10]. Although the biological importance of lncRNA as a variety of transcripts has been revealed, its natural function and potential as a drug target for stroke remain largely undefined.

Autophagy belongs to caspase-independent programmed death, which is the phagocytosis and degradation of foreign bodies, damaged or aged organelles in the cytoplasm by the autophagy-lysosomal system. Autophagy activation is a double-edged sword that plays an important role in brain I/R injury and will affect the fate of neurons that suffer from cerebral ischemia/reperfusion [11, 12]. Recent studies suggest that acute and severe ischemia may lead to “excessive autophagy” that promotes cell death and damage. LncRNAs generally modulate autophagy via regulating the expression of autophagy-related genes, such as ATGs [13]. They often act as competing endogenous RNAs (ceRNAs) to regulate microRNAs associated with autophagy [14, 15]. Therefore, combined intervention via lncRNA and autophagy may be a promising therapeutic approach.

Here, using an RNA-Seq sequencing analysis of lncRNA profiling in ischemic hemisphere from a tMCAO/reperfusion mouse model, we identified lncRNA PEG11as (PEG11as) as an upregulated lncRNA. Further, the bioinformatic analysis revealed that PEG11as, which is located on the antisense strand corresponding to mouse chromosome 12qF1 protein encoding gene Rtl1 (retrotransposon Gag like 1, Rtl1), and the transcription length is 1315bp. But up to now there is no evidence for a role for PEG11as in stroke. Bioinformatics analysis revealed that PEG11as, as a ceRNA, competes with miR-874-3p whose binding sites are enriched in autophagy genes. However, whether the PEG11as is involved in ischemic stroke via sponging with miR-874-3p, especially regulating cerebral ischemia-induced autophagy, remains unknown.

Therefore, it is hypothesized that up-regulated PEG11as directly sponge with miR-874-3p to inhibit miR-874-3p activity, leading to excessive activation of autophagy, which in turn leads to brain damage. PEG11as silencing significantly inhibits autophagy hyperactivation and ameliorates brain neuronal damage, suggesting PEG11as as a stroke biomarker or therapeutic target.

## MATERIALS AND METHODS

### Animals

C57/BL6 mice (♂, ± 28.0 g) were purchased from the Hubei Provincial Center for Disease Control and Prevention (SCXK (Q) 2015–0018). The proposed experiments and research protocols were assessed and approved by the experimental ethics committee of Shanghai University of Medicine and Health Sciences.

### Intracerebroventricular (ICV) injection

PEG11as siRNA lentivirus (shRNA-PEG11as) and its negative-EGFP lentivirus (shRNA-NEG) were injected intracerebroventricularly (a rate of 1 μL/min in a 4 μL total volume) after mice were anesthetized with 10% chloral hydrate [16] (Supplementary Table 1).

### A mouse MCAO model

Mice were anesthetized (body temperature maintained at 37 ± 0.3ºC) using only 30% and 69% 1–2% isoflurane-oxygen/nitrous oxide mixtures. The left common carotid artery, external carotid artery, and internal carotid artery were isolated and guided with a silicone-coated 6–0 suture from the external carotid stump to the internal carotid artery until reaching the middle cerebral artery lumen. Vascular embolism was assessed with a laser Doppler flowmeter (Moor Instruments, UK). Sham-operated animals used the same procedure except that no embolization was performed [17].

### Neurological evaluation

Neurological assessment was performed using the methods of Clark score and modified neurological severity score (mNSS) after 24 hours of reperfusion. Clark score was correlated with underlying infarct volume [18]. mNSS test assesses overall neurological function, including assessment of motor responses, sensation, reflexes and balance, according to the guidelines of Chen et al. [19].

### 2, 3, 5-triphenyltetrazolium chloride (TTC) staining

TTC staining was used to evaluate cerebral infarct volume [20]. Briefly, 24 hours after mouse perfusion, each mouse was anesthetized, and brain tissue was rapidly removed and cut into 2 mm slices in the coronal direction. Each slice was incubated in 2% TTC for 30 minutes and then fixed with a 4% paraformaldehyde solution. The infarct volume was assessed accordingly: volume of the ischemic region (pale)/total volume × 100%.

### Transmission electron microscope analysis

The hippocampal CA1 region was isolated after perfusion with glutaraldehyde. The samples were fixed with 4% paraformaldehyde for 2 h and embedded in epoxy resin. Cut the tissue into 90 nm ultrathin sections. Autophagic vacuoles were observed using a transmission electron microscope (HZ600, Hitachi, Japan).

### Terminal deoxynucleotidyl transferase dUTP nick-end labeling (TUNEL) staining

The ischemic hemisphere was sectioned, stained with the TUNEL detection kit system, and the cell morphology was observed with a laser scanning confocal microscope (CX31-32RFL, Olympus, Tokyo, Japan). The percentage of the infarct volume was measured using Image J software (ver. 1.61). The percentage of the brain-infarct volume was calculated as infarct volume/total volume × 100%.

### Real-time RT-PCR

Total RNA was extracted with Trizol reagent. Reverse transcription was performed using 2 μg of total RNA according to the guidelines of the First Strand cDNA Synthesis Kit (Thermo Fisher Scientific, K1622, Waltham, MA, USA). The expression of PEG11as, miR-874-3p, β-actin, and U6 (Ribobio, Guangzhou, Guangdong, China) was analyzed using SYBR Green Real-time PCR Master Mix (Takara, Shiga, Japan). Initially, denaturation was conducted at 95°C for 2 min, followed by 40 cycles, including denaturation at 95°C for 10 s, annealing at 60°C for 20 s, and elongation at 70°C for 20 s. Primers used in this study are listed in Supplementary Table 1. The lncRNAs level was normalized to β-actin, and the miR-874-3p level was normalized to U6.

### Cell culture and the treatment of OGD/R

Mouse neuroblastoma Neuro-2a (N2a) cells were purchased from the Cell Bank of the Chinese Academy of Sciences (Shanghai, China) and cultured in Dulbecco’s Modified Eagle Medium (DMEM) containing 10% fetal bovine serum (Gibco, USA) and 1% antibiotics (100 U/mL penicillin and 100 μg/mL streptomycin) at 37°C in a humidified 5% CO_2_ incubator. To simulate ischemia/reperfusion (I/R) *in vitro*, the OGD/R model was applied as described above [21]. Briefly, cells were inoculated in 6-well plates with a cell density of 3 × 10^5^ per well. After 24 h, the normal medium was refreshed with the glucose-free DMEM (Gibco, USA), and then placed in a box containing a mixture of gas of 95% N2 and 5% CO_2_ at 37°C for 3 h. Thereafter, the glucose-free medium was replaced by a complete DMEM. The control group was treated identically however they were not exposed to OGD.

### Cell transfection

Cells were seeded in 6-well plates and transfected with a PEG11as siRNA lentivirus or transduced with a mimic or inhibitor of miR-874-3p or a negative control (Shanghai Genechem Co., Ltd., China). After 48 hours, the inhibitory effect of LV-siRNA-PEG11as was detected, which was used for the next experiment.

### Western blot

Ischemic tissues and cells were kept in ice-cold RIPA buffer with 0.1 mM phenylmethylsulfonylfluoride (PMSF) (Sigma, St. Louis, MO, USA) to extract the total proteins. The resolved proteins were transferred to polyvinylidene fluoride (PVDF) membrane under the condition of 300 mA for 1.5 h, and then incubated at room temperature for 2 hours in a TBST buffer (20 mM Tris-HCl, pH 7.6,150 mM NaCl, 0.1% Tween-20) containing 5% no-fat milk. Specific primary antibodies were incubated at 4°C overnight, including ATG16L1 (Cell Signaling Technology, 8089S), SQSTM1/p62 (Servicebio, GB11531), GAPDH (Proteintech, 60004-1-Ig) and LC3A/B (Servicebio, GB11124). Further, the PVDF membrane was incubated with corresponding HRP-conjugated secondary antibodies for 90 min at room temperature. Blots were visualized by an enhanced chemiluminescent (ECL) reagent (Amersham Biosciences, Piscataway, USA) under a Bio-Rad ChemiDoc MP system (Bio-Rad, Hercules, CA, USA). Bands were quantified through Image J.

### Cell viability

N2a cells were seeded into 96-well plates at a density of 8 × 10^3^ cells per well. After 24 h of the transfection of LV-PEG11as-shRNA or its negative vector, Cells underwent 4 h of OGD and reperfusion for 0, 6, 12, 24 and 48 hours, respectively. Add 20 μl of 3-(4,5-dimethylthiazol-2-yl)-2,5-diphenyltetrazolium bromide (5 mg/mL) to each well and resuspend at 37°C in the dark. Removing the supernatant, the crystalline formazan was added 150 μl of dimethyl sulfoxide to dissolve. Absorbance was measured at 490 nm (Omega Bio-tek, Norcross, GA, USA).

### Nissl staining

Brain slices were deparaffinized in xylene and dehydrated in a gradient of 70, 75, 90, 95, and 100% in ethanol and stained with thionine for 1 hr at 37°C. The neuronal morphology in cerebral cortex was observed to assess brain injury under a microscope (Olympus, Japan) [22].

### Luciferase reporter gene assay

The mutated (MUT) and wild-type (WT) sequences of the 3′-UTR segments of PEG11as and ATG16L1 containing the putative miR-874-3p binding sites were constructed and cloned into the pmiR- RB-REPORT vector (OBiO Technology Corp., Ltd, Shanghai, China) with pmirGLO to generate PEG11as-WT, PEG11as-MUT, ATG16L1-WT, and ATG16L1-MUT luciferase reporter. The luciferase activity was measured using a luciferase assay kit (Promega, Madison,WI, USA) after transfection with the luciferase reporter plasmids for 48 h [23].

### Fluorescence *in situ* hybridization (FISH) analysis

Cy3-labeled probes were synthesized for FISH, which was used to detect PEG11as expression in cells. Briefly, cells were fixed with 4% formaldehyde for 10 min at room temperature and then prehybridized in 0.5% TritonX-100-PBS at room temperature. The FISH probes were hybridized in a hybridization buffer in the dark at 37°C overnight, the probe sequence was TGGAGTACCCTCGAGTGGAGATGAGGATCCTTCCAATCCGGGCTGCCTTCATGGTGTGGTGCCGCTACCTGGAGAACACCGAGGAGCCCATCATGATCCTTCTCAACACAGAGGATCTAGCCTCTCTGAATAATGACAGGCTCACCGTACTTCTCCCCGGGCATTGGGTCTTCTTCTTCTCACACTTCAATTTTGGTGTTATGGAGATGCCAGCTGAAGGTGAC. Nuclei were counter-stained with 4,6-diamidino-2-phenylindole (DAPI) after multiple washes with saline-sodium citrate (SSC) buffer. PEG11as detected under confocal laser microscopy (CX31-32RFL, Olympus, Tokyo, Japan).

### Microarray analysis in a MCAO/R mouse model

The three samples of ischemic penumbra obtained from the tMCAO/R mice for the microarray experiments were performed by RiboBio Co., Ltd. (Guangzhou, China) [21]. Total RNA was extracted from cells using the TRIzol kit (Invitrogen, Carlsbad, CA, USA). High-throughput sequencing was performed using Agilent Feature Extraction from OE BioTech (Shanghai, China). Differentially expressed LncRNAs in brain tissues were screened by genome-wide microarray expression profiling, |fold change|>2, adjusted *P* < 0.05.

### Autophagy flux assay

N2a cells were treated with mCherry-GFP-LC3 adenovirus (C3011, Beyotime Biotechnology) at 20 MOI (multiplicity of infection) at 37°C and 5% CO_2_/95% air. After 24 h, the virus-containing medium was removed and then transduced with or without LV-PEG11as, a mimic or inhibitor of miR-847-3p, or its negative control, and then exposed to OGD for 4 h/R for 24 h. Immunofluorescence was detected by confocal laser scanning microscopy [24]. Under a fluorescence microscope (Olympus), count the number of GFP and mCherry spots per cell in three randomly selected fields.

### Statistics

All results were expressed as mean ± SEM. The data was analyzed using a *t*-test, one-way ANOVA. The *t*-test was used to compare the differences between the two groups, whereas the one-way ANOVA was used to compare the differences among multiple groups.

## RESULTS

### PEG11as is upregulated in cerebral IS/R and cellular OGD/R

**Figure 1:**
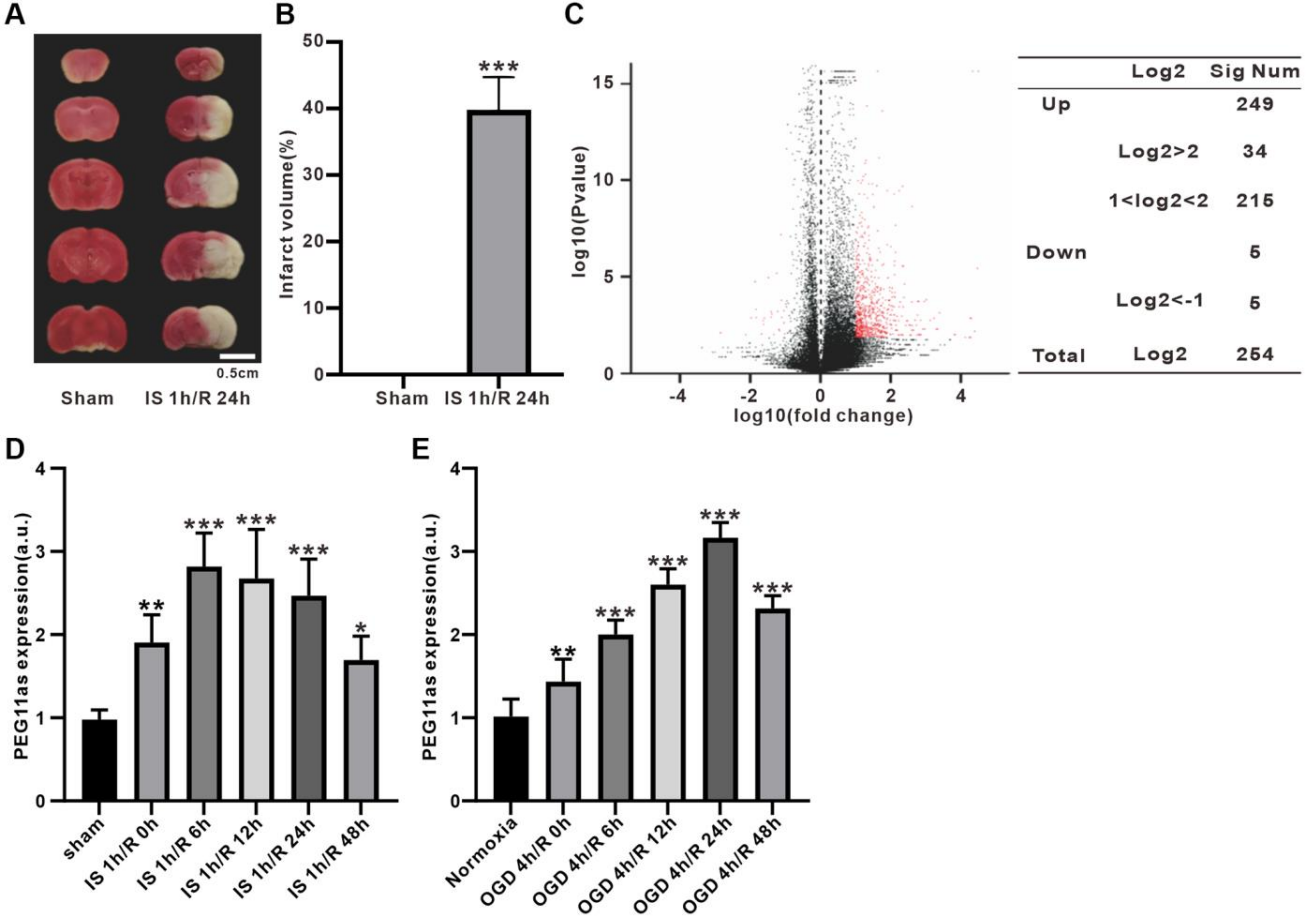
**PEG11as expression in tMCAO/R mice and cellular OGD/R models.** (**A**, **B**) TTC staining evaluation and statistical analysis of mouse cerebral infarct volume induced by MCAO 1 h/R 24 h (*n* = 6). (**C**) Volcano plot of lncRNA expression differences in ischemic penumbra. Red spots represent differentially expressed genes. (**D**, **E**) PEG11as level determined by RT-PCR in the ischemic penumbra of cerebral IS/R mice (*n* = 6) and OGD/R-treated N2a cells (*n* = 6). One-way ANOVA followed by the Tukey’s post-hoc-test was used, data are shown as mean ± SD. ^*^P < 0.05, ^**^P < 0.01 and ^***^P < 0.001 vs. sham group.

A microarray of lncRNA expression was performed in a tMCAO/R mouse model. Compared to sham group, infarct volume increased significantly in IS/R group (Figure 1A, 1B). The lncRNA expression profile showed that 254 lncRNAs (254 up-regulated/5 down-regulated) were significantly different in the ischemic penumbra expressed after tMCAO/R treatment (|log2(Fold change) |>1, adjusted *P* < 0.05) (Figure 1C). Of these, PEG11as expression was significantly up-regulated, as verified by RT-PCR in tMCAO/R mice and OGD/R-treated N2a cells (Figure 1D, 1E) (Supplementary Table 2).

### PEG11as silencing reduces acute neuronal damage in tMCAO/R mice

**Figure 2:**
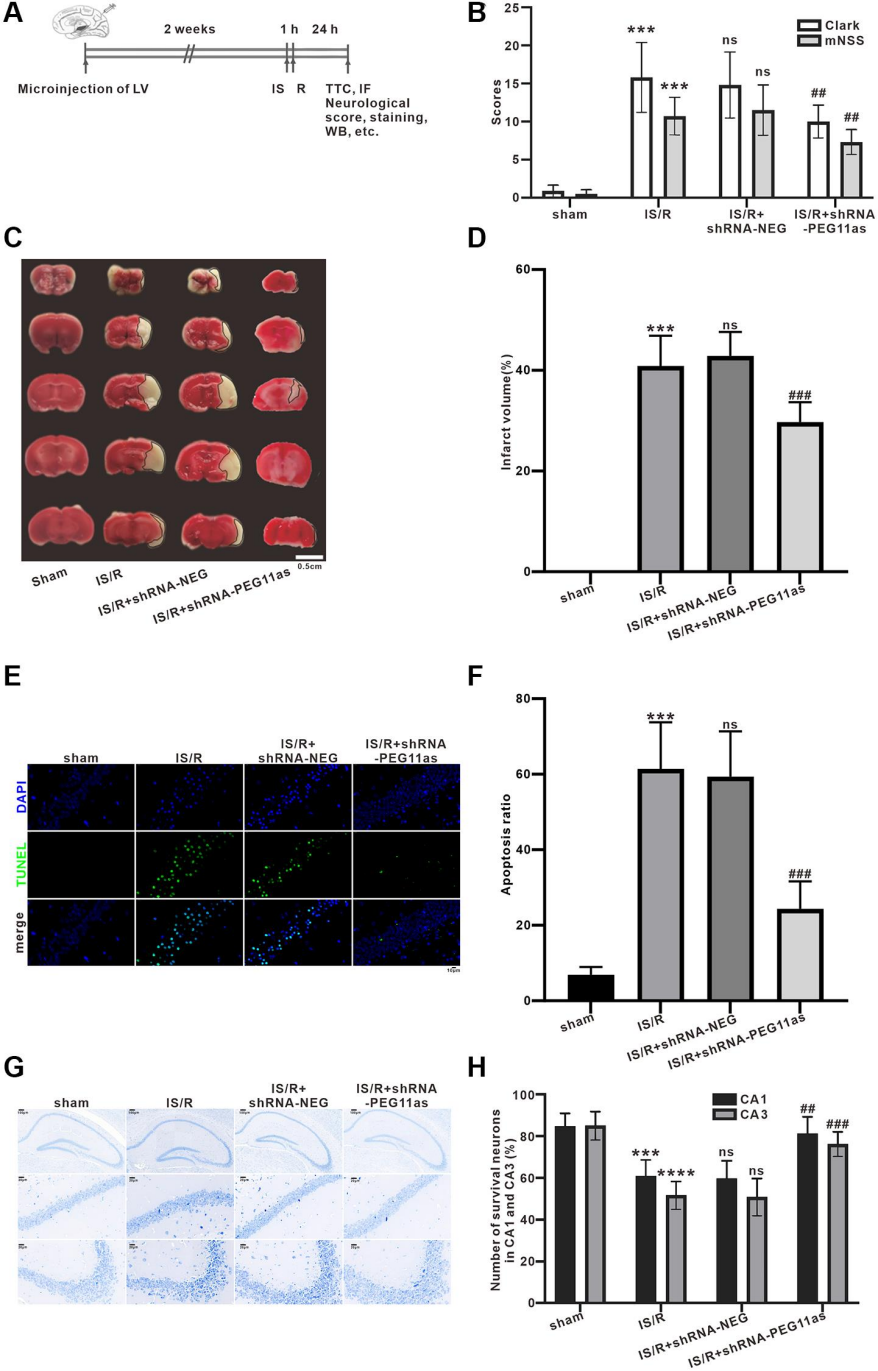
**PEG11as silencing alleviates the acute injury of cerebral neurons induced by cerebral IS/R.** (**A**) Illustration of the experimental procedure. (**B**) The average neurological scores of Clark and mNSS tests (*n* = 10). (**C**, **D**) Representative pictures and statistical chart for TTC staining. (**E**, **F**) Representative images and statistics of TUNEL staining of brain sections for assaying neuronal apoptosis. (**G**, **H**) Representative pictures and statistical chart of Nissl staining in the CA1 and CA3. *n* = 6. One-way ANOVA followed by the Tukey’s post-hoc-test was used, data are shown as mean ± SD. ^***^P < 0.001 vs. sham group, ^#^*P* < 0.05 or ^##^*P* < 0.01 vs. IS/R+shRNA-NEG group. Abbreviation: ns: no statistically different vs. IS/R group.

To determine the role of PEG11as in cerebral IS/R insult, a PEG11as siRNA lentivirus (shRNA-PEG11as) and its negative (shRNA-NEG) were constructed. After 2 weeks of intracerebroventricular injection of shRNA-PEG11as or shRNA-NEG, mice were subjected to MCAO for 1 hour followed by 24 hours of reperfusion (Figure 2A). According to the mNSS results, the IS/R group had obvious neurological deficits compared with the sham group, which was significantly alleviated by injection of shRNA-PEG11as (Figure 2B). Clark score results also presented similar results (Figure 2B). Meanwhile, significant cerebral infarction was found in IS/R group than that in sham group, while PEG11as silencing reduced infarct volume compared to IS/R+shRNA-NEG group (Figure 2C, 2D). In addition, TUNEL analysis found that the number of TUNEL-positive cells in IS/R group was significantly higher than that in sham group, while the number of positive cells was significantly reduced after shRNA-PEG11as injection (Figure 2E, 2F). Similar protective effects were found in the results of Nissl staining. Treatment of shRNA-PEG11as obviously reduced the number of Nissl bodies compared with the shRNA-NEG+IS/R group (Figure 2G, 2H). The above data revealed that PEG11as silencing inhibited cerebral IS/R-induced neuronal damage.

### PEG11as silencing inhibits autophagy activation and neuronal apoptosis *in vivo*

**Figure 3:**
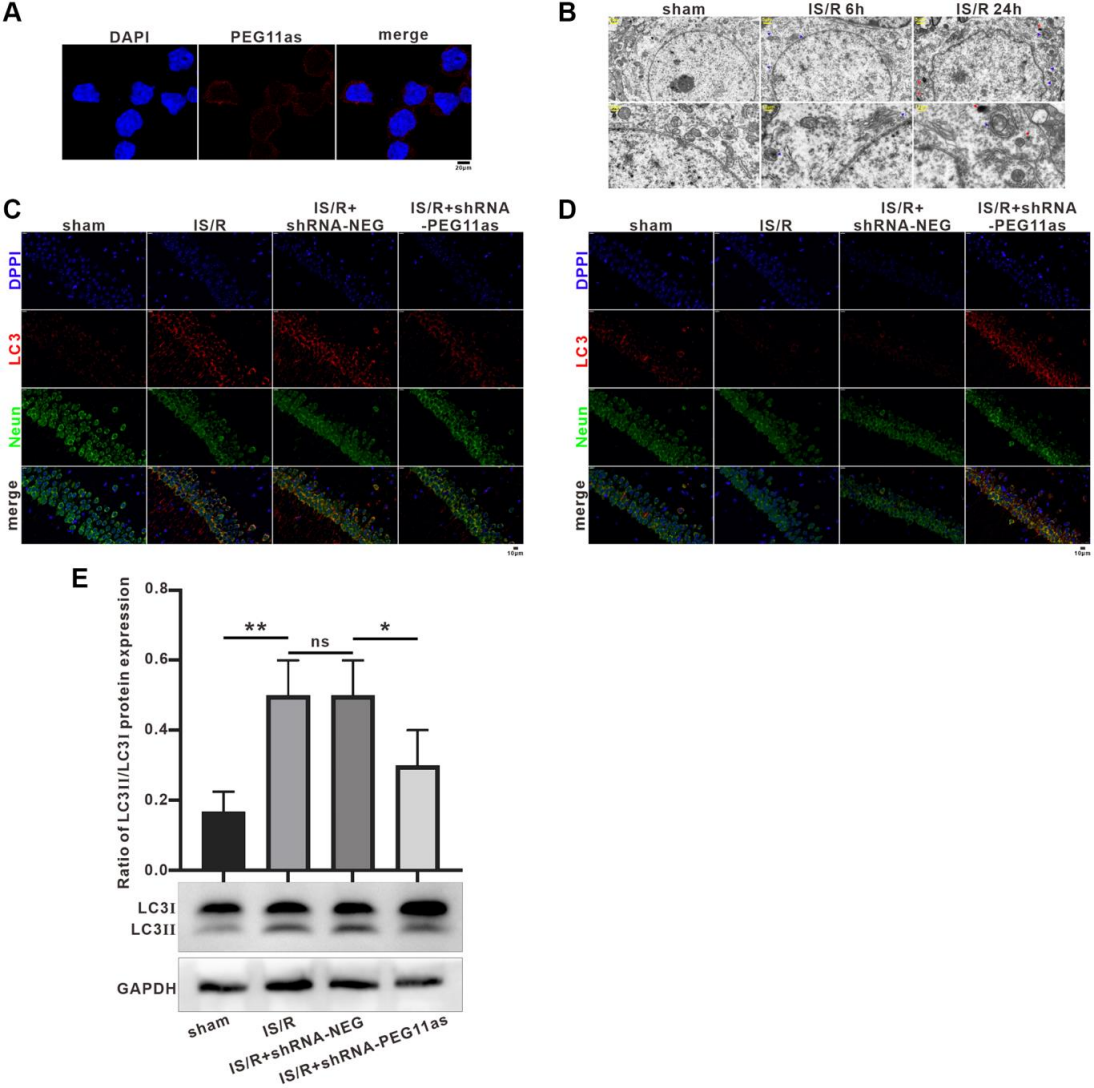
**PEG11as silencing inhibited neuronal autophagy and apoptosis.** (**A**) A FISH assay showed the location of PEG11as in mouse primary neurons. Green, PEG11as; Blue, DAPI. (**B**) Transmission electron microscopy was applied to observe the ultrastructural features in hippocampal of tMCAO/R mice. Blue arrow indicated autophagosomes and yellow arrow represented lysosomes. (**C**, **D**) Representative double immunofluorescent staining for NeuN and MAP1LC3B (**C**) and SQSTM1/p62 (**D**) in ischemic hemispheres transfected with shRNA-PEG11as 14 days and treated with tMCAO/R. (**E**) Representative pictures and statistical chart for the western blot of MAP1LC3 staining. *n* = 3. One-way ANOVA followed by the Tukey’s post-hoc-test was used, data are statistically different from each other with ^*^P < 0.05, ^**^P < 0.01 and ^***^P < 0.001. Abbreviation: ns: no statistically different vs. IS/R group.

Given the critical role of PEG11as in stroke *in vivo*, we next wanted to explore its role in this process. We first detected PEG11as expression in N2a cells using fluorescence in situ hybridization (FISH). The results showed that PEG11as was mainly expressed in the cytoplasm (Figure 3A), suggesting that PEG11as may compete with endogenous RNA by binding to miRNA through complementary base pairing. Kyoto encyclopedia of genes and genomes (KEGG) was used to speculate PEG11as potential functions via the candidate miRNA-mRNA (http://www.genome.jp/) (Supplementary Figure 1). Target gene-enriched KEGG classes including ‘autophagy’ and ‘mTOR signaling pathway’ have been involved in ischemia stroke [25]. These results predicted the biological process of autophagy might be involved in the function of PEG11as on cerebral IS/R.

TEM showed an increase in the number of autophagosomes in the cytoplasm after MCAO, and both lysosomes and autophagosomes increased significantly with prolonged reperfusion time (Figure 3B), consistent with previous studies [26]. In addition, the effect of PEG11as on the expression of autophagy-related molecules in the MCAO stroke model was investigated. Immunohistochemistry analysis indicated that MAP1LC3B expression were significantly induced in IS/R and IS/R+shRNA-NEG mice (Figure 3C), which was reversed by microinjection of PEG11as siRNA. Meanwhile, microinjection of PEG11as siRNA significantly rescued the depletion of p62 compared with that in IS/R or IS/R+shRNA-NEG group (Figure 3D). Consistently, western blot showed that the increasing of MAP1LC3B-II level induced by cerebral IS/R was downregulated compared with IS/R+shRNA-NEG mice (Figure 3E).

### PEG11as is a target of miR-874-3p

**Figure 4:**
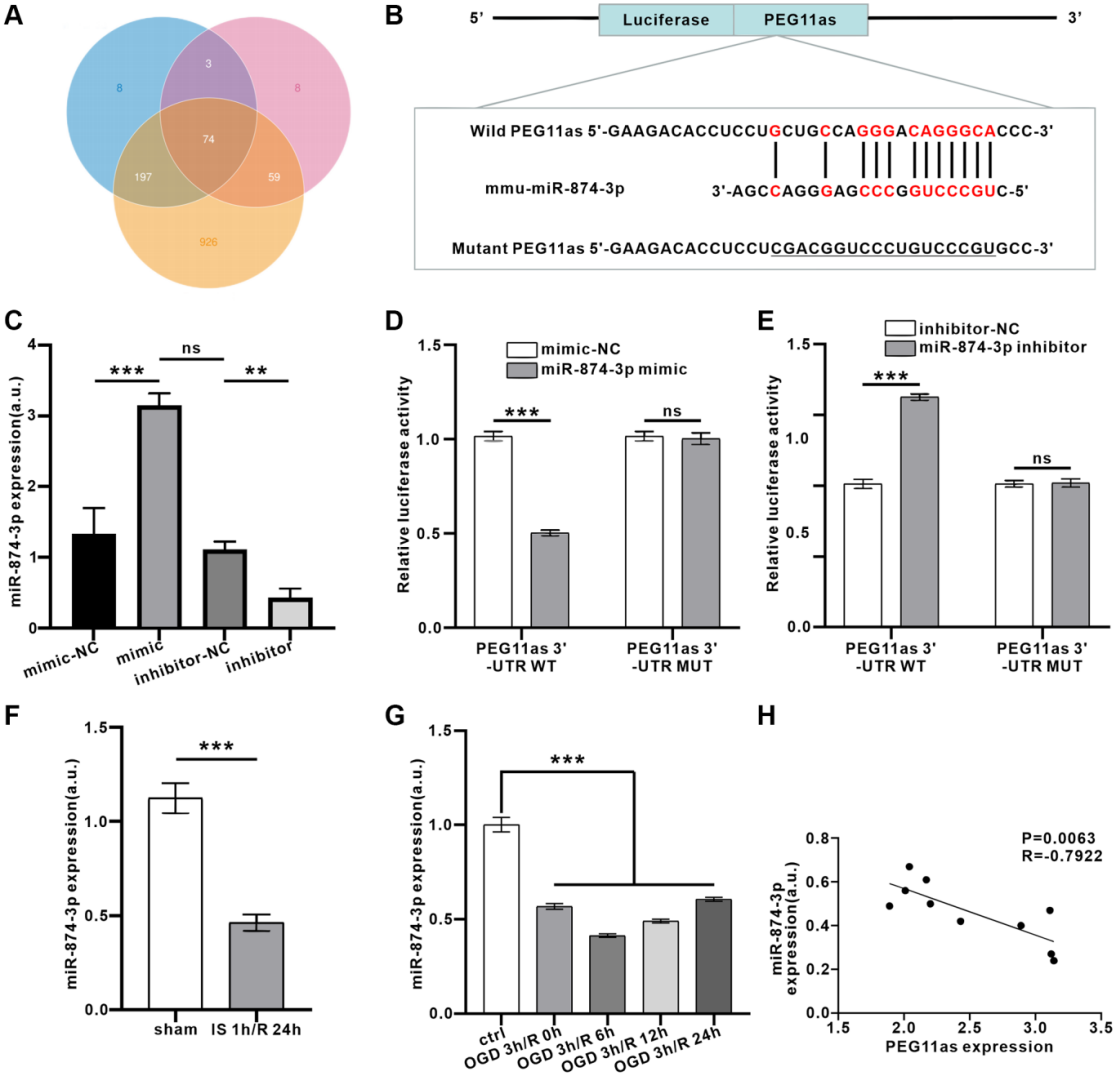
**PEG11as bound to miR-874-3p and reduced miR-874-3p expression.** (**A**) Predicted binding miRNAs of PEG11as derived from 3 databases. (**B**) Binding sites between PEG11as and miR-874-3p predicted in the web-based bioinformatic software. (**C**) N2a cells were transfected with miR-874-3p mimic or miR-874-3p inhibitor and their mimic-NC or inhibitor-NC. After 48 h, RT-PCR was utilized to detect the miR-874-3p expression. *n* = 3 in each group. (**D**, **E**) The luciferase reporter vector carrying PEG11as-WT (**D**) or PEG11as-MUT (**E**) was co-transfected with NC mimic or miR-874-3p mimic or NC inhibitor or miR-874-3p inhibitor. 48 hours later, the relative luciferase activity was measured. *n* = 3 in each group. (**F**, **G**) miR-874-3p expression in the mouse brains treated by MCAO/R (**F**) or in N2a cells treated by OGD/R (**G**). *n* = 6 (*in vivo*) or *n* = 3 (*in vitro*). (**H**) The negative correlation between the expression levels of PEG11as and miR-874-3p by performing qRT-PCR in the mouse brains treated by MCAO/R. One-way ANOVA followed by the Tukey’s post-hoc-test was used, data are shown as mean ± SD. Data are statistically different from each other with ^*^P < 0.05, ^**^P < 0.01, and ^***^P < 0.001. Abbreviation: ns: no statistically different vs. IS/R group.

Based on *in vivo* studies showing that PEG11as is involved in autophagy activation, the next step is to clarify its molecular mechanism. LncRNAs affect target protein expression when endogenous RNA sponges interact with miRNAs [21]. We analyzed candidate miRNAs interacted with PEG11as by the software of miRanda, PITA and RNAhybrid. The intersection results of lncRNA-miRNA interaction in the three software were shown in Venn diagram. As shown in Figure 4A, a total of 74 miRNAs were predicted. Among them, miR-874-3p exhibited high scores, speculating that PEG11as competed with miR-874-3p as a ceRNA. Bioinformatic analysis was used to predict the binding site between PEG11as and miR-874-3p (Figure 4B). To examine the ability of PEG11as to bind to miR-874-3p, mimics and inhibitors of miR-874-3p were constructed. 48 h after transfection of these sequences, miR-874-3p was successfully overexpressed or knocked down (Figure 4C). Further, upregulation of miR-874-3p expression reduces luciferase activity in PEG11as-WT but not PEG11as-MUT (Figure 4D). Conversely, silencing miR-874-3p increased luciferase activity in PEG11as-WT, but had no significant effect on luciferase activity in PEG11as-MUT (Figure 4E), implying that miR-874-3p bound to PEG11as at the forecasted binding site. Furthermore, treatment with IS 1 h/R 24 h *in vivo* (Figure 4F) or with OGD 4 h/R 24 h *in vitro* (Figure 4G) abolished compared to control group miR-874-3p expression, indicating the opposite pattern of PEG11as expression (Figure 4H).

### ATG16L1 may be a target of miR-874-3p

**Figure 5:**
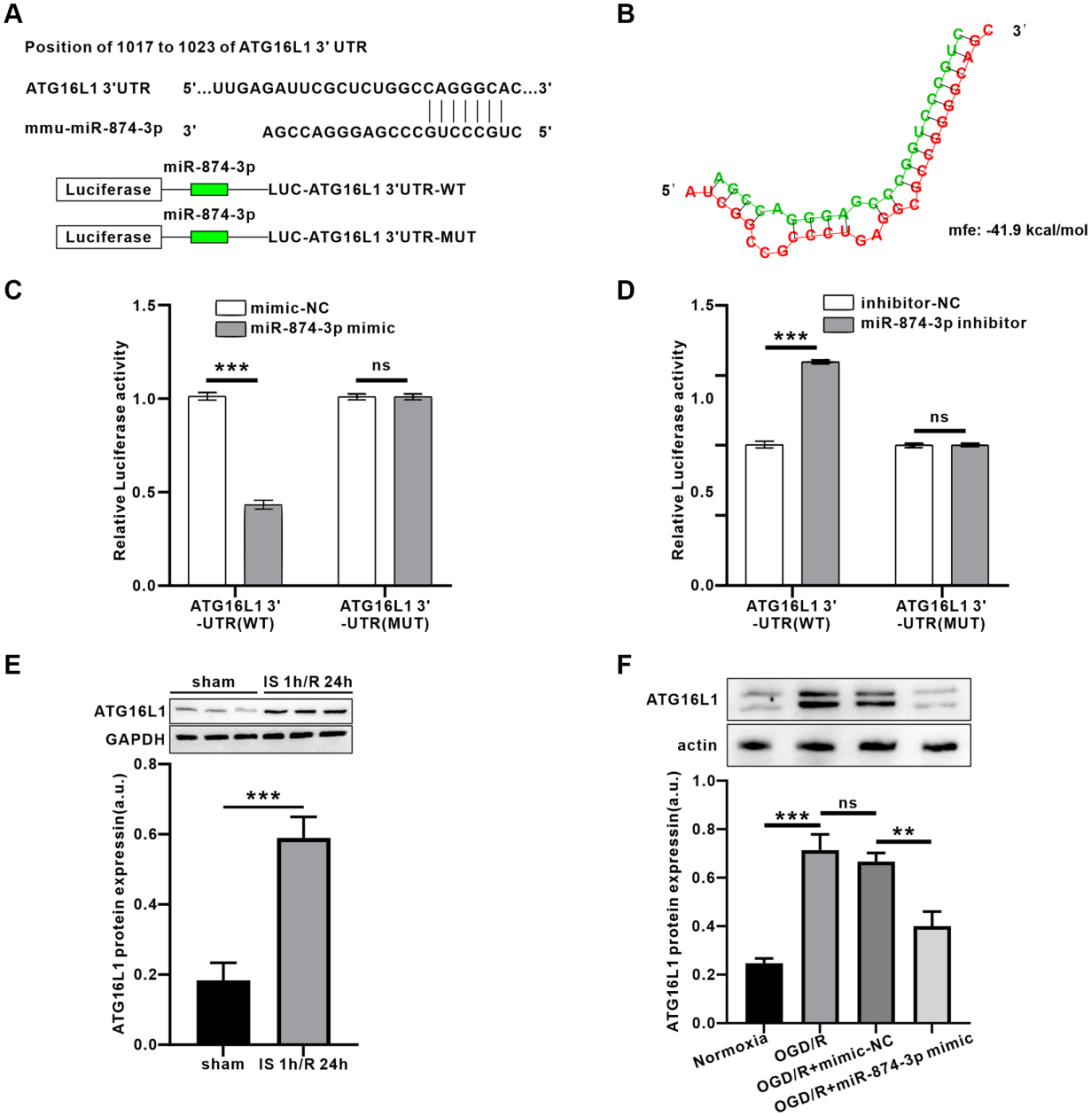
**PEG11as positively regulated ATG16L1expression by sponging miR-874-3p.** (**A**) Possible binding sites of miR-874-3p in DDIT4 3’-UTR. (**B**) Bibiserv2 software was used to analyze 3’-UTR Hybrid energy analysis between miR-874-3p and ATG16L1. (**C**, **D**) The ATG16L1 3’-UTR-WT or ATG16L1 3’-UTR-MUT was determined after co-transfected with miR-765 mimic (**C**) or miR-874-3p inhibitor (**D**). (**E**) ATG16L1 protein level analyzed by western blot in the mouse brains treated by MCAO/R. (**F**) ATG16L1 protein level analyzed by western blot in N2a cells transfected with miR-874-3p mimic. One-way ANOVA followed by the Tukey’s post-hoc-test was used, data are shown as mean ± SD. Data are statistically different from each other with ^*^P < 0.05, ^**^P < 0.01, and ^***^P < 0.001.

The target genes of miR-874-3p were predicted using Targetscan. Interestingly, we found a complementary pairing area between the sequences of miR-874-3p and ATG16L1 in the 3′UTR of Mus musculus (mmu) (Figure 5A). Further, 3′-UTR mixed energy analysis showed that the free energy between miR-874-3p and ATG16L1 was lower (−41.9 kcal/mol) using Bibiserv2 software, suggesting that the two may have strong binding ability (Figure 5B). Luciferase reporter gene analysis revealed that miR-874-3p directly targeted ATG16L1 through a putative binding site (Figure 5C, 5D, *P* < 0.01). In addition, the expression of ATG16L1 in IS 1 h/R 24 h or OGD 4 h/R 24 h groups was significantly higher than that in sham or control groups. Furthermore, transduction of miR-874-3p mimic reduced ATG16L1 expression compared with the mimic-NC group (Figure 5E, 5F).

### PEG11as silencing inhibits OGD-induced neuronal autophagy and apoptosis via miR-874-3p/ATG16L1 axis

**Figure 6:**
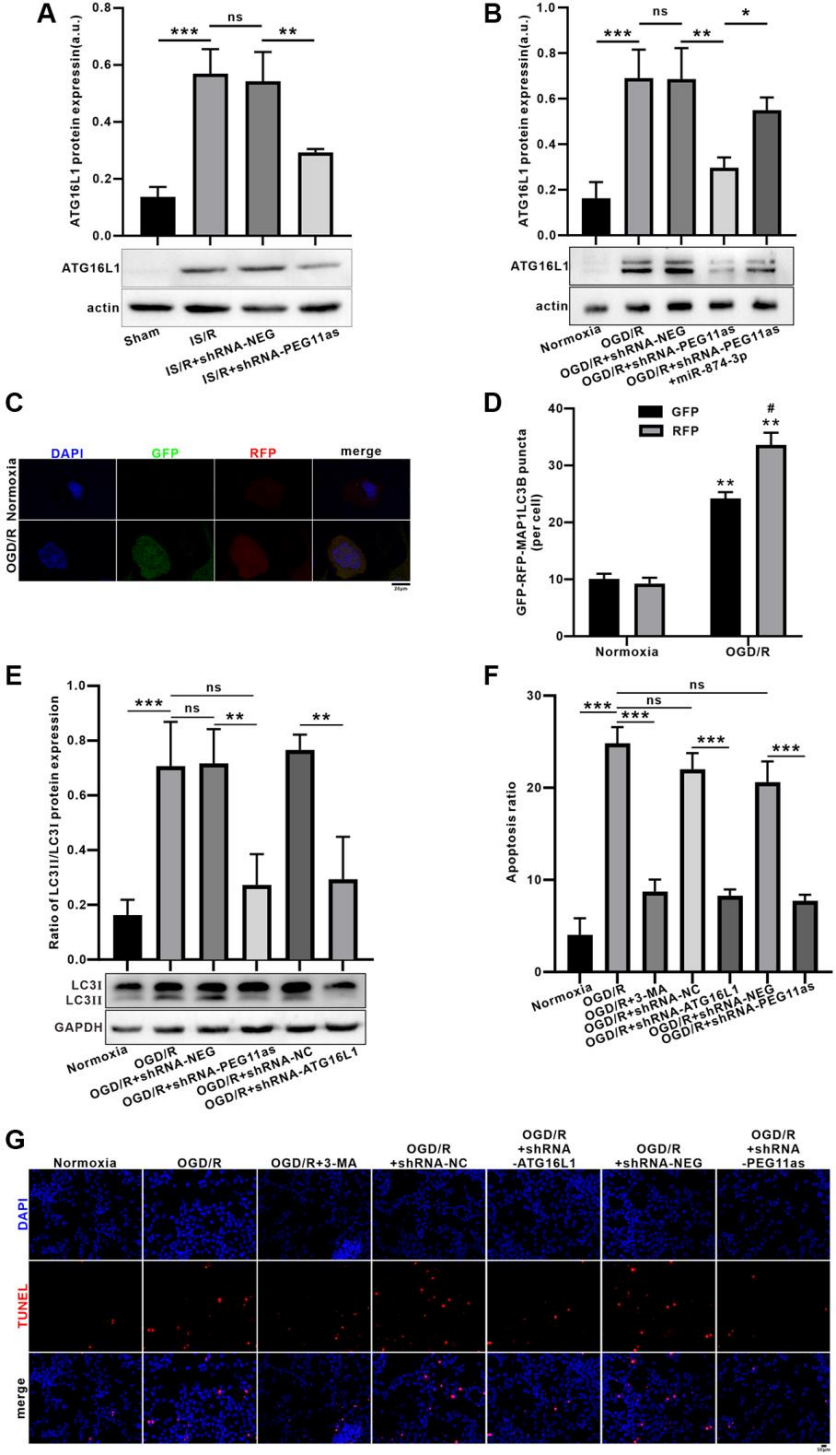
**Inhibition of PEG11as alleviated OGD/R-induced apoptosis by ameliorating autophagic flux defects via miR-874-3p/ATG16L1 axis in N2a cells.** (**A**) PEG11as silencing down-regulated ATG16L1 expression in tMCAO/R mice after transfection with sh-PEG11as. *n* = 6 animals/group. (**B**) PEG11as regulated ATG16L1 expression by sponging miR-874-3p. The cells were transfection with sh-PEG11as or co-treated with miR-874-3p inhibitor. 48 hours later, the cells were exposed to OGD 4 hours and 24 hours of Reperfusion in N2a cells. *n* = 3. (**C**, **D**) Knockdown of ATG16L1 ameliorate OGD/R-induced autophagic flux defects. (**E**) LC3-II/LC3-I ratio and p62 level were determined by western blot after transfection of ATG16L1 siRNA in N2a cells exposed to OGD/R. (**F**, **G**) TUNEL staining for analysis of the cell apoptosis after treated with 3-MA, sh-PEG11as or sh-ATG16L1, respectively. *n* = 3. One-way ANOVA followed by the Tukey’s post-hoc-test was used, data are shown as mean ± SD. Data are statistically different from each other with ^*^P < 0.05, ^**^P < 0.01, and ^***^P < 0.001.

MCAO/R significantly upregulated ATG16L1 expression the protein levels; however, microinjection of shRNA-PEG11as lentivirus obviously abolished the increasing of ATG16L1 level in tMCAO/R mice (Figure 6A). Consistent with the *in vivo* findings, OGD 4 h/R 24 h treatment increased ATG16L1 expression in N2a cells. However, shRNA-PEG11as transduction significantly suppressed ATG16L1 levels in OGD/R-treated N2a cells (Figure 6B). To further illustrate that miR-874-3p interacts with PEG11as to regulate ATG16L1 levels, N2a cells were co-transduced with miR-874-3p inhibitor and shRNA-PEG11as. Anti-miR-874-3p reverses shRNA-PEG11as-induced downregulation of ATG16L1 expression (Figure 6B).

Furthermore, autophagic flux assays demonstrated a significant increase in the number of yellow puncta per cell in the OGD/R-treated group, while transduction of tandem fluorescently labeled mRFP-GFP-MAP1LC3B increased RFP-only MAP1LC3B puncta (Figure 6C, 6D). To explore the effect of PEG11as or ATG16L1 on autophagy activation, autophagy levels were assessed using MAP1LC3 expression. Co-transduced with PEG11as siRNA, ATG16L1 siRNA or its negative control, the increasing of MAP1LC3B-II/LC3B-I ratio was found in OGD/R group; however, the increase effect was reversed by transduction of PEG11as siRNA or ATG16L1 siRNA (Figure 6E). In addition, incubation of N2a cells with 3-methyladenine (3-MA; 600 nmol, intracerebroventricularly) inhibited OGD/R-induced apoptosis. As shown in Figure 6F, 6G, 3-MA significantly decreased the apoptosis rate, supporting its protective effect via inhibition of autophagy. Further, treatment with shRNA-ATG16L1 or shRNA-PEG11as also inhibited the apoptosis induced by OGD/R, suggesting that knockdown of PEG11as or ATG16L1 has a neuroprotective effect via inhibiting autophagy (Figure 6F, 6G).

## DISCUSSION

The mechanism of neurological damage caused by stroke has not been fully elucidated. Despite decades of efforts to find neuroprotective agents against cerebral ischemia, for example, genetic approaches have recently been proposed for stroke treatment, using viral vectors carrying transcription factors such as Sox2, Ngn2, or Ascl1 to isolate astrocytes which direct reprogramming into neurons [27], but further research is needed.

Altered lncRNA expression in the post-stroke brain has been studied using microarrays [28, 29] and RNA-Seq [30]. These early studies provide some preliminary evidence for the functional of lncRNAs in stroke. While lncRNA profiling has been demonstrated in MCAO/R and OGD-R models, to our knowledge, our study is the first to identify the lncRNA PEG11as in a tMCAO mouse stroke model using RNA-Seq sequencing. We further elucidated the effect of PEG11as-miR-874-3p-ATG16L1 axis involved in the vivo tMCAO/R and vitro OGD/R models, in particular, its effect in neuronal autophagy and whether this effect contributed to its protection in neuronal damage, suggesting that PEG11as may act as an important mediator involved in brain I/R-activated autophagy-activated.

LncRNAs play specific regulatory roles at transcriptional, post-transcriptional and epigenetic stages, are highly specific in CNS, and can effectively regulate central nervous system development and disease progression [31]. This study established a correlation between PEG11as and brain IS/R. In addition, silencing PEG11as inhibits IS/R on neuronal damage and improves neurological dysfunction. These data hinted that the correlation of PEG11as and the pathogenesis of stroke and its protection on ischemic stroke.

LncRNAs, acting as ceRNAs to regulate target gene expression via interacting with microRNAs [32]. FISH data showed that PEG11as was mainly distributed in the cytoplasm. Thus, bioinformatics was used to evaluate PEG11as function as a ceRNAs [33]. As previous findings, it was predicted that the biological process of autophagy might be involved in the function of PEG11as on cerebral IS/R. MAP1LC3B/LC3B is induced by autophagy activation to cleave LC3-phosphatidylethanolamine conjugates [34]. SQSTM1/p62 acts as a receptor to link LC3B-II to the ubiquitin moiety on misfolded proteins, mediating clearance of ubiquitinated proteins [35]. The data showed that PEG11as silencing inhibited IS/R-induced autophagy over-activation, along with reversing the increasing of MAP1LC3B/LC3B-II expressions, and the decreasing of SQSTM1/p62 expression induced by cerebral ischemia/R, hinting that knockdown of PEG11as expression inhibited the autophagic dysfunction.

Based on the proof that PEG11as is involved in autophagy activation, the next step is to clarify its molecular mechanism. Bioinformatics analysis and luciferase activity assay predicted and further confirmed the fact that PEG11as competes with miR-874-3p as a ceRNA. Furthermore, *in vitro* and *in vivo* studies showed that the expression of miR-874-3p exhibited an opposite pattern to that of PEG11as.

The ATG12-ATG5-ATG16L1 and LC3 protein complex plays an important role in autophagosome formation [36]. ATG12-ATG5-ATG16L1 mediates lipidation by recruiting ATG3-ATG8 to the membrane and promoting transfer to phosphatidylethanolamine (PE) [37]. Interestingly, a complementary pairing region between miR-874-3p and ATG16L1 sequences was found in the 3′UTR of Mus musculus (mmu), and the software predicted its binding energy to be strong via Bibiserv2 software, which was confirmed by luciferase reporter gene analysis, indicating ATG16L1 was target of miR-874-3p directly. In addition, ATG16L1 levels were induced up-regulated by I/R and OGD/R, and this effect was reversed by PEG11as silencing. Moreover, the role of ATG16L1 in autophagy activation was also examined. Apparently ATG16L1 siRNA inhibits autophagy activation and rescues over digestion of autophagy substrates.

Growing evidence suggests that autophagy play an important role in ischemic stroke [13, 38, 39]. The interplay between autophagy and apoptosis appears to determine cell fate. It is proposed that autophagy is beneficial only if autophagic flux is intact and may cause deleterious effects if impaired autophagic flux primarily results in cell death. Furthermore, moderate activation of autophagy appears to be beneficial for stroke; however, excessive activation of autophagy suggests a pro-death effect [40]. It is clarified that ischemic excitotoxicity is involved in autophagic flux. Pretreatment with 3-MA inhibits autophagy at an early stage of autophagosome formation, decreased OGD-R-induced neuronal apoptosis. It has been proven that knockdown of ATG16L1 significantly rescue autophagy defect. Moreover, it also inhibited neuronal apoptosis. So, we then sought to examine the effect of PEG11as silencing in OGD/R-induced neuronal apoptosis. As expected, PEG11as siRNA inhibited neuronal apoptosis induced by OGD/R in N2a cells. Our current study suggest that autophagy enhances neuronal apoptosis, while PEG11as silencing demonstrated the neuroprotection might be partially dependent on ATG siRNA-induced autophagy inhibition.

In conclusion, this study reveals the underlying molecular mechanism of brain I/R injury-induced PEG11as upregulation, suggesting that the PEG11as-miR-847-3p-ATG16L1 axis is involved in I/R injury-induced autophagy activation. Studies highlight the role of PEG11as in ischemia-induced autophagy and attribute its protection to inhibition of autophagy hyperactivation. However, more in-depth studies are needed to elucidate the role of PEG11as in ischemia, including its regulation of ischemic injury-induced autophagy, which will provide insights into the management and treatment of ischemic stroke, especially for stroke patients outside the treatment window. Therefore, our study may provide a possible interventional target for the treatment of stroke.

## Supplementary Materials

Supplementary Figure 1

Supplementary Tables
