# Supplementary material for: LncRNA PEG11as silencing sponges miR-874-3p to alleviate cerebral ischemia stroke via regulating autophagy in vivo and in vitro
Source: Aging (Albany NY). 2022 Jun 24;14(12):5177–94. doi: 10.18632/aging.204140 (PMC9271312; doi:10.18632/aging.204140)
Supplement: Supplementary Figure 1 [file aging-14-204140-s001.pdf]

SUPPLEMENTARY FIGURE

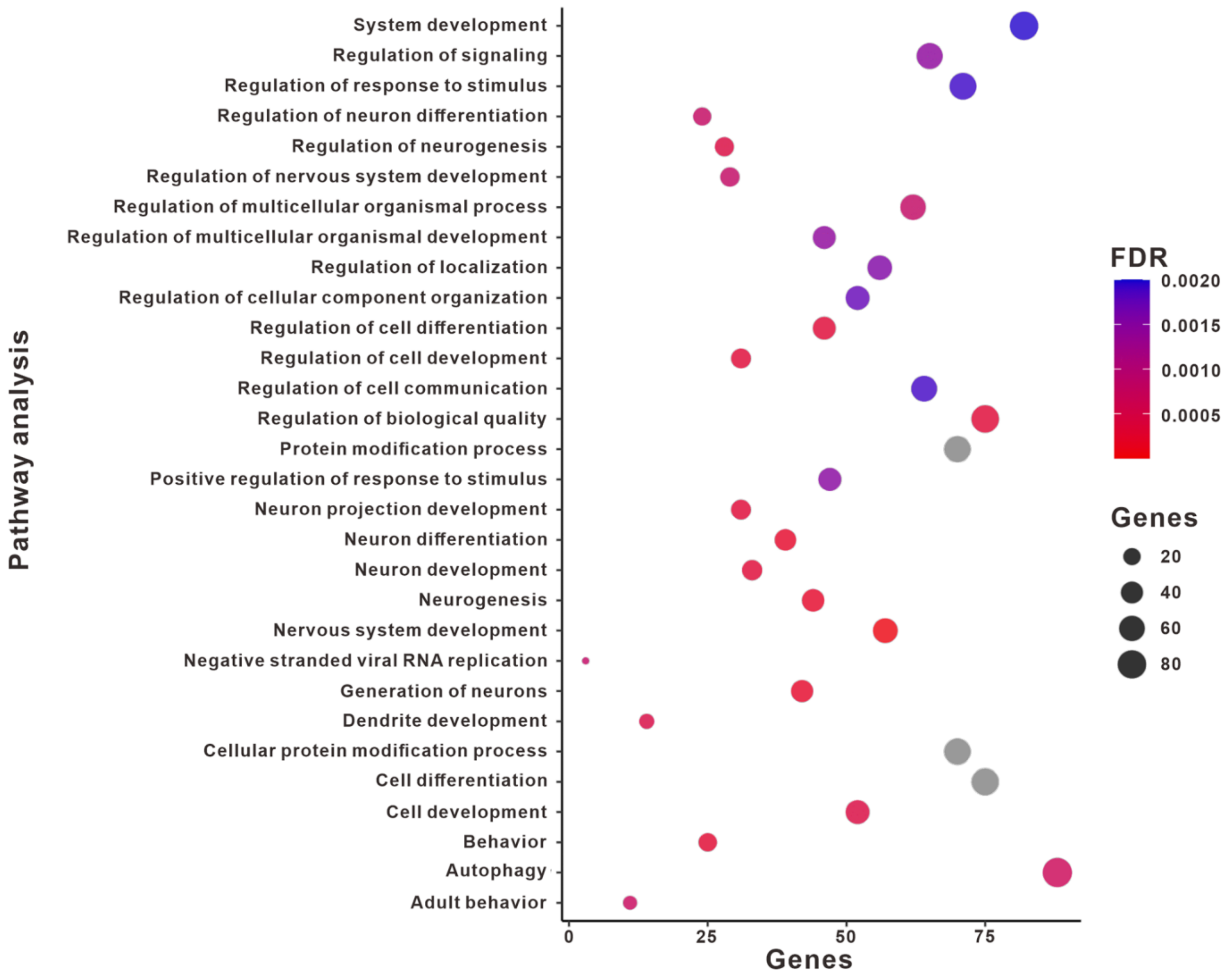

Supplementary Figure 1. The analysis of Kyoto encyclopedia of genes and genomes (KEGG) of PEG11as.
