# Supplementary material for: LncRNA PEG11as silencing sponges miR-874-3p to alleviate cerebral ischemia stroke via regulating autophagy in vivo and in vitro
Source: Aging (Albany NY). 2022 Jun 24;14(12):5177–94. doi: 10.18632/aging.204140 (PMC9271312; doi:10.18632/aging.204140)
Supplement: Supplementary Tables [file aging-14-204140-s002.pdf]

SUPPLEMENTARY TABLES

Supplementary Table 1. Sequences.

Name	Sequence
Mouse PEG11as F	5′TGATCCTGGGGAGCTACGAA3′
Mouse PEG11as R	5′TTGAGCAACTTCCACGCTCA3′
Mouse PEG11as shRNA1	TAGGATGAAGTGAACAATGTT
Mouse PEG11as shRNA2	GAGGTTCATAAGGTTCTTGTT
Mouse PEG11as shRNA3	CAGGTAGATTTCTTGCCAGTT
Mouse negative-shRNA	TTCTCCGAACGTGTCACGT
Mouse ATG16L1-shRNA1	5′GAAUGAUAGUCAACUACAATT3′
	5′UUGUAGUUGACUAUCAUUCTT3′
Mouse ATG16L1-shRNA2	5′CCAAGUUCCUGCUGGACAATT3′
	5′UUGUCCAGCAGGAACUUGGTT3′
Mouse ATG16L1-shRNA3	5′GAGAUAAAUGUGAAUUCAATT3′
	5′UUGAAUUCACAUUUAUCUCTT3′
Mouse miR-847-3p mimic	5′CUGCCCUGGCCCGAGGGACCGA3′
Mouse miR-847-3p inhibitor	UCGGUCCCUCGGGCCAGGGCAG
Mouse NC mimic	5′UUGUACUACACAAAAGUACUG3′
Mouse inhibitor mimic	5′CAGUACUUUUGUGUAGUACAA3′

Supplementary Table 2. Partially differentially expressed LncRNAs.

Gene	Sham	I/R	log_2FoldChange	*p*-value
fantom3_7330403C04	3.681696667	83.2402	4.498837758	2.22E-16
fantom3_F730006E08	0.115788367	2.350246667	4.343249963	2.22E-16
fantom3_D830013O18	1.052845333	4.797543333	2.188002323	0
fantom3_D330015J21	0.506524	2.072308333	2.032536141	0
fantom3_A230063K01	0.481251333	1.527642	1.666444047	6.85E-12
fantom3_9530008I07	0.396179333	1.223912667	1.627275088	2.22E-16
C030018K13Rik	0.397606333	1.15321	1.53624261	8.89E-05
fantom3_D930039P20	0.372825333	1.068771667	1.519381868	2.33E-09
PEG11as	3.613243333	10.04659	1.475339586	0
fantom3_6430411K18	3.613243333	10.04659	1.475339586	0
fantom3_5330411G14	2.27501	6.192653333	1.444684799	0
fantom3_0610011H19	0.525776533	1.391748333	1.404376698	0.00011997
fantom3_F630301H07	1.357556667	3.464893333	1.35179852	0
fantom3_C230053O12	0.500025	1.22446	1.292073513	9.22E-12
fantom3_9430062P13	0.421794667	1.021389667	1.27592061	1.79E-07
fantom3_3830422K02	0.577392	1.392575667	1.270132696	1.28E-11
fantom3_C330024I08	0.578894333	1.374666667	1.247709892	5.32E-10
fantom3_B930049N16	0.681532333	1.613326667	1.243184577	2.56E-07
fantom3_1700037N05	0.529379667	1.228629667	1.214675437	6.22E-06

fantom3_A630050J02	0.635355333	1.45032	1.190735678	9.83E-11
fantom3_B230216K14	0.601096667	1.362237667	1.180309504	0
fantom3_6530425O17	0.654239	1.47754	1.17530752	4.86E-06
fantom3_4932703K07	1.18434	2.624186667	1.147787038	1.36E-10
fantom3_B230208E11	0.468363	1.021311333	1.124723705	5.69E-06
fantom3_F930050L08	1.075358667	2.339916667	1.121639226	0
fantom3_B930059N03	0.564166667	1.225846667	1.1195852	6.62E-09
fantom3_A130068E22	1.338923333	2.900306667	1.115132099	0
fantom3_1520401O13	0.998430667	2.151556667	1.107646686	1.66E-08
fantom3_2210010H06	0.711999	1.533926667	1.107282393	7.69E-07
fantom3_4732477A13	0.546897333	1.170616667	1.097926792	5.05E-10
fantom3_6030408D07	0.517050667	1.089982333	1.075927187	2.64E-07
fantom3_C130034A22	1.155198333	2.404213333	1.057424351	0
fantom3_C130012C18	0.498973	1.036618	1.054850693	3.04E-06
fantom3_D330050J08	0.611742333	1.26245	1.045230229	3.50E-07
fantom3_D030040M07	1.359993333	2.804133333	1.04395537	0
fantom3_D930015A08	0.604673333	1.245877	1.042933783	5.38E-08
fantom3_5330416G15	0.55117	1.131821333	1.038076966	3.84E-05
fantom3_9030205G03	0.830252667	1.686163333	1.022121936	1.94E-06
fantom3_A930017D03	0.675015333	1.360651	1.011304891	1.33E-06
Gm11627	3.21384	1.464252333	−1.134133913	6.42E-08
